# Current tests for diagnosis of hepatitis B virus infection and immune responses of HBV-related HCC

**DOI:** 10.3389/fonc.2023.1185142

**Published:** 2023-11-28

**Authors:** Wanting Shi, Kang Li, Yonghong Zhang

**Affiliations:** ^1^Interventional Therapy Center of Liver Disease, Beijing You'An Hospital, Capital Medical University, Beijing, China; ^2^Biomedical Information Center, Beijing You'An Hospital, Capital Medical University, Beijing, China

**Keywords:** hepatitis B virus (HBV) infection, hepatocellular carcinoma, immune responses, diagnosis, management

## Abstract

Chronic hepatitis B virus (HBV) infection is a worldwide public health threat that results in huge morbidity and mortality. Late diagnosis and delayed treatment of HBV infections can cause irreversible liver damages and occurrence of cirrhosis and hepatocellular carcinoma (HCC). Detection of the presence and activity of HBV are the cornerstones of diagnosis and management in HBV related disease. Moreover, comprehensive knowledge of the mechanisms regulating HBV immunobiology is pivotal for managing diseases related with HBV. Here we tried to categorize and illustrate the classical and novel approaches used for diagnosis of HBV. Also, we reviewed our current knowledge on the immunobiology of HBV related HCC.

## 1 Introduction

Hepatitis B virus (HBV) infection is a major health problem, with the total worldwide HBV infection prevalence increased to 3.9% and 2 billion people infected globally (1). For about 257 million people with chronic infection, only approximately 10% were diagnosed with HBV infection (2). The major life-threatening events for patients with chronic hepatitis B (CHB) are the development of liver cirrhosis and oncogenesis (3–5). In 2021, World Health Organization (WHO) indicated that primary liver cancer is the sixth most common global malignancy and the third highest in mortality (6). The direct (viral genome associated) and indirect (host immune response associated) mechanisms in the pathogenesis of liver cancer has been discussed (7), among which immune imbalance plays a pivotal role in HBV-related cancer development. These complications for patients with CHB typically occur after several years of persistent low-level liver disease characterized by hepatocellular regeneration and inflammation. Therefore, early diagnosis and proper management are of great significance for patients with CHB. Understanding the mechanism of these HBV markers induced dysregulation of immune function leading to complications is also necessary. Here we tried to categorize and illustrate the classical and novel approaches used for diagnosis of HBV. Also, we reviewed our current knowledge on the immunobiology of HBV related hepatocellular carcinoma (HCC).

## 2 Venus blood test

### 2.1 Serological testing

HBV-specific antigens and antibodies appeared after infection are imperative serologic markers to diagnose and categories the phases of HBV infection, therefore guide treatment principle. At present, diverse serological diagnostic assays are utilized, comprising rapid diagnostic tests (RDTs) and laboratory-based immunoassays including enzyme immunoassays (EIAs), chemiluminescence immunoassays (CLIAs), and electrochemiluminescence immunoassays (8).

The most widely used screening test for HBV is the hepatitis B surface antigen (HBsAg), while the test for evaluation of immunity post recovery or vaccination is hepatitis B surface antibody (HBsAb). The disappearance of serum HBsAg could indicate immune control and long-time inhibition of HBV replication. Qualitative detection of HBsAg is a common method for screening and diagnosis of HBV infection. Quantitative HBsAg (qHBsAg) measurement has been employed to discriminate inactive carriers with very low risk of reactivation and proposed as a useful complement to HBV DNA load for treatment monitoring in chronic HBV infection patients (9–11). Quantifying HBsAg can be used as a valuable tool in management of patients receiving pegylated interferon (peg-IFN) therapy (11), including response prediction and deciding the time to start or terminate treatment (12). Monitoring qHBsAg during nucleos(t)ide analogue (NA) treatment can help identify patients who will achieve or sustain inactive status or develop HBsAg clearance upon discontinuation of treatment (13). For HBeAg-negative patients with HBV DNA levels < 2000 IU/mL, follow-up intervals can be determined according to whether baseline levels of HBsAg are lower than 1000 IU/mL in EASL guidelines.

At present, there are mainly two approaches for detection of qHBsAg. The ARCHITECT^®^ HBsAg QT is a CLIA and Roche Diagnostics Elecsys HBsAg II collects signals via electrochemiluminescence. Lumipulse HBsAg-HQ, with 10-fold higher sensitivity than that of conventional assays, is currently used as a high-sensitive automatic HBsAg assay (detection limit: 5 mIU/ml) (14). Moreover, an ultra-sensitive HBsAg assay based on a semi-automated immune complex transfer chemiluminescence enzyme immunoassay (ICT-CLEIA) with detection limit of 0.5 mIU/ml has been developed (**Table 1**) (15).

**Table 1:** New biomarkers for Hepatitis B virus infection.

HBV diagnostic markers	Method	Assay	Characteristics (LOD)	Interpretation	Clinical application
Quantitative HBsAg	CLIA	The ARCHITECT^®^ HBsAg QT	Conventional assay (50 mIU/mL)	Marker of infected hepatocytes and transcriptionally active cccDNA	1. Identify inactive carriers 2. Manage CHB patients receiving antiviral therapy 3. Decide follow-up intervals
	ECL	Elecsys HBsAg II	Conventional assay (50 mIU/mL)		
	CLEIA	Lumipulse HBsAg-HQ	High-sensitive automatic assay (5mIU/ml)		
		ICT-CLEIA	Ultra-sensitive, semi-automated ICT assay (0.5 mIU/ml)		
HBcrAg	CLEIA	Lumipulse G-HBcrAg	Conventional assay with lower sensitivity in HBeAg-negative patients (sensitivity: 2.1 Log U/ml)	Marker of intrahepatic cccDNA and its transcription	1. Monitor the progression of CHB 2. Evaluate the effectiveness of NA therapy when DNA replication is markedly suppressed 3.Early detection of HBV reactivation 4.Predict the development of complications
		iTACT-HBcrAg;	Fully automated, high-sensitivity assay for HBeAg-negative patients (sensitivity: 2.8 Log U/ml)		
Quantification of HB core antibody	ELISA	Beijing Wantai Biological	Sandwich-based enzyme-linked immunosorbent assay	Marker of HBV core antigen-specific immune activity and HBV exposure	1. Predict occult HBV infection 2. Identify responders to anti-HBV therapies. 3. Predict HBeAg seroconversion rate
	CLEIA	Lumipulse G HBcAb-N	Fully automated two-step sandwich technology (0.5 IU/mL)		
HBV NRAg	ELISA	HBV NRAg; Beijing Wantai Biology	ELISA based assay to detect PreS1 antigen and HBV core antigen qualitatively	Marker of viral replication and highly correlated with cccDNA activity	1. Associate with higher rates of liver abnormalities 2. Surrogate for HBV DNA quantification in low resource area
HBV RNA	PCR	Cobas^®^ 6800/8800 investigational HBV RNA assay	High throughput, sensitive and inclusive assay of HBV RNA quantification	Transcript derived from HBV cccDNA during replication	1. More accurate reflection of cccDNA transcription activity 2. Monitor antiviral efficacy when HBV DNA is supressed 3. Predict viral recurrence when NA is stopped 4.Predict the occurrence of hepatocellular carcinoma
		HBV-SAT kit Rendu biotechnology, Shanghai, China	RNA simultaneous amplification testing (100copies/mL)		
		Abbott m2000 RNA RUO assay	High throughput test with greater sensitivity than rapid amplification of complementary DNA ends analysis (45 U/L)		

CLIA, chemiluminescence immunoassay; ECL, electrochemiluminescence; CLEIA, chemiluminescence enzyme immunoassay; HBV, hepatitis B virus; ICT, immune complex transfer; CHB, chronic hepatitis B; ELISA, enzyme-linked immunosorbent assay; HBsAg, hepatitis B surface antigen; HBcAb, hepatitis B core antibody; PCR, polymerase chain reaction; HBcrAg, hepatitis B core-related antigen; HBeAg, hepatitis B virus e antigen; NA, nucleos(t)ide analogue; cccDNA, covalently closed circular DNA; HBV NRAg, HBV nucleic acid related antigen.

The hepatitis B core antigen (HBcAg) and hepatitis B e antigen (HBeAg) is encoded by the precore and core regions of core gene respectively. HBeAg, indicating HBV replication and infectivity, is a truncated HBcAg containing the precore region. The existence of HBeAg in the serum of an HBsAg-positive carrier implies frequent viral replication and greater infectivity. The emergence of anti-HBe and loss of HBeAg during acute self-limited infection predict clearance (16). Detecting HBeAg and anti-HBe is crucial in determining the stage of CHB infection (17), which correlates with active HBV replication and elevated HCC risk. Commercial quantitative tests for HBeAg are not available. With regard to the quantification of HBeAg, published data are limited. Smilar to other serum markers, HBeAg levels might contribute to track disease course, predict HBeAg seroconversion and evaluate response to anti-HBV therapies.

Hepatitis B core-related antigen (HBcrAg) comprises HBcAg, HBeAg and a truncated core-related protein p22cr, sharing an identical 149 amino acid sequence (18). Hepatitis B core (HBc) IgM and HBc IgG are respectively utilized to discriminate acute and chronic infections. The concentration of HBcrAg correlates with HBV DNA copies in serum and the intrahepatic covalently closed circular DNA (cccDNA) copies (19). HBcrAg has been applied to monitor the progression of CHB and predict outcomes (20, 21). In HBeAg negative patients with CHB, HBcrAg might help monitor the effectiveness of NA therapy when HBV DNA replication is markedly suppressed. In a large cohort of HBV Japanese patients, HBcrAg level >3 Log U/ml was tested as an independent risk factor for the occurrence of HCC (22). A traditional HBcrAg detection method (Lumipulse G-HBcrAg [G-HBcrAg]; Fujirebio, Inc.) uses the CLEIAs (23). Recently, a high sensitive automatic CLEIA for HBcrAg detection (iTACT-HBcrAg; Fujirebio, Inc, Tokyo, Japan) has been reported, which exhibited about 10-fold more sensitive than the traditional assay (24).

Lacking neutralizing ability, HB core antibody (Anti-HBc) represents HBV exposure sensitively and can be used to predict occult HBV infection (OBI) (25). Anti-HBc IgG value was correlated with the presence of intrahepatic HBV cccDNA (26). Some ELISA-based assays (anti-HBc ELISA, Beijing Wantai Biological; Lumipulse G HBcAb-N, Fujirebio) have been commercially developed to quantify anti-HBc (qAHBc) (27). This could serve as a valuable surrogate to anticipate the likelihood of OBI reactivation among immunosuppressed patients (26). Elevated levels of anti-HBc might signify a robust immune response against HBV, which helped predict the response to treatment and the risk of relapse after stopped treatment (28). Higher baseline qAHBc was related with a greater HBeAg seroconversion rate in HBeAg-positive patients receiving either NA or peg-IFN treatment.

Pre S1 protein and core antigen of hepatitis B virus particles are markers of virus replication, collectively referred to as HBV nucleic acid related antigen (HBV NRAg). A new approach using ELISA (HBV NRAg; Beijing Wantai Biology, Beijing, China) has been reported to detect PreS1 antigen and HBcAg qualitatively. PreS1 antigen has demonstrated the alternative reflect of HBV replication and cccDNA activity (29, 30).

### 2.2 Molecular diagnostics

HBV DNA. HBV DNA, a direct marker of HBV genome replication, is applied to evaluate therapy efficacy and guide treatment (17, 31, 32), especially for patients on NAs therapy. HBV DNA levels at baseline or during treatment can also predict the response to peg interferon (peg IFN) treatment (33, 34). The high level of HBV DNA is closely related to the occurrence of cirrhosis and HCC, therefore antiviral therapy mainly targets HBV DNA (35, 36). Inhibiting HBV DNA to an undetectable level during detection is related to lower risk of cirrhosis, decompensation and HCC (37). Real-time PCR is used for the current HBV DNA quantitative method with excellent analytical performance, including the detection limit and the wide linear range of 10-100 million IU/mL.

cccDNA. The host enzymes utilize cccDNA as a template for transcription, resulting in the production of new virions (38). The existence of cccDNA in the nucleus of hepatocyte infection is the key reason why HBV is difficult to eradicate (12). Liver biopsy is required for the measurement of cccDNA and the standardization of quantification still has problems in clinical practice. Several serum markers have been considered to surrogate cccDNA, including HBV DNA, HBV RNA, serum HBcrAg and qHBsAg.

HBV RNA. Serum HBV RNA is mainly composed of HBV pre-genomic RNA (pgRNA) which is a transcript derived from HBV cccDNA during replication (39). Other forms of serum HBV RNA detected low content includes spliced variants of pgRNA, precore RNA, HBx transcripts and truncated forms (40, 41). As an effective alternative indicator reflecting the transcription activity of cccDNA, HBV RNA can cover the “blind spot” of HBV DNA and HBsAg to monitor antiviral efficacy especially when treatment suppress HBV DNA significantly (42). Since the suppressed HBV DNA levels constantly fail to reflect the levels of viral cccDNA for patients on NA therapy, elevated pgRNA might predict viral recurrence when NA is stopped (39). The serum HBV RNA predicts HBeAg seroconversion on NA or peg IFN therapy (43, 44). Detectable HBV pgRNA and DNA were correlated with 2-year risk of HCC development and the former is a stronger risk factor for predicting the occurrence of HCC (45). Ding et al. also confirmed that serum pgRNA could be used to predict the prognosis and recurrence of HCC after hepatectomy (46).

The detection of HBV RNA in serum is mainly based on quantitative reverse transcription-polymerase chain reaction (RT-qPCR): rapid amplification of cDNA ends-based RT-qPCR (47), standard RT-qPCR, droplet digital PCR (ddPCR) (48) and reverse transcriptase ddPCR (49). In 2022, the cobas^®^ 6800/8800 investigational HBV RNA assay with high throughput, sensitive and inclusive was reported to assess the clinical relevance of HBV RNA quantification (50). The HBV-SAT kit (Rendu biotechnology, Shanghai, China) uses the HBV RNA simultaneous amplification detection method (HBV-SAT) based on fluorescence detection of RNA transcription-mediated nucleic acid amplification (51). Abbott m2000 real-time PCR system (Abbott Molecular, Des Plains, Illinois) was applied in commercial HBV RNA measurement through PCR technology. However, because of the sophistication and expensiveness, it is currently challenging to utilize these techniques in areas with limited resources.

Genotype Testing and drug resistance. There are 10 HBV genotypes from A to J with different geographical distribution, which are classified according to the divergence of HBV genomic nucleotide sequence of 8% or higher (52). The four predominant genotypes are A to D (53). Different genotypes are related with persistence of viral load, risk of developing cirrhosis and HCC, HBsAg seroclearance, drug resistance, as well as prognosis (54, 55). Although initial diagnosis dose not mandate HBV genotype testing, sequencing of genotypes and drug resistance mutations can be helpful to predict the individual response probability and should be tested when considering peg IFN treatment according to the AASLD and EASL guidelines (12, 17, 56).

The gold standard approach for HBV genotyping involves conducting whole genome sequencing and subsequent phylogenetic analyses (57, 58). Other systems include multiplex nested PCR or real-time PCR, reverse hybridization, restriction fragment polymorphism (RFLP), oligonucleotide microarray chips, reverse dot blot, restriction fragment mass polymorphism and invader assay (58).

The occurrence of a specific mutation in hepatitis B virus might lead to drug resistance, which is significant for management of HBV-infected patients. Early and appropriate detection of drug resistance indicates a therapeutic modality change (58). Sequence-based assays are used for detection of HBV drug resistance mutations. The Sanger sequencing is acknowledged as a “gold standard”. Real-time PCR, RFLP, hybridization and high-throughput next-generation sequencing are also applied to detect drug resistance mutations (55).

## 3 Diagnose HBV using dried blood spot

Requiring little staff training, RDTs are simple, low cost, and allow usage of variable specimen types (e.g. venous, fingerstick blood, and oral fluid). RDTs offered at point-of-care (POC) are needed for difficult-to-access populations (59). In the past few years, significant advances have been made in POC tests.

Dried blood spot (DBS) specimens have achieved satisfying results due to the low cost, avoidance of venipuncture, easier handling, transportation with lower biohazard risk, and convenient storage options (60). HBV antigen, antibodies and DNA could be detected by EIA and PCR method in DBS and the diagnostic accuracy and precision have been confirmed by many studies (61, 62). In 2022, the applicability of DBS for sequencing, as HBV genotyping and tracking mutations was evaluated (63). The 2017 WHO Guidelines recommended that DBS should be considered for serological and nucleic acid testing HBV and HCV technologies where laboratory detection is not available or when patients showed poor venous access (8). Despite its benefits, limitations exist including the lower sensitivity of detection, interference from disrupted cellular constituents in DBS drying process and difficulty to adapt commercial tests to be used on DBS samples (64–66). Strict validation and assessment of sensitivity and specificity are necessary for DBS diagnosis (66).

## 4 Diagnose HBV using oral fluid

The diagnosis with oral fluid samples is simple and noninvasive to achieve in children and other patients with difficult venous access. Commercial enzyme immunoassay and PCR method in oral fluid could detect HBV antigen, antibodies, and DNA (66). The efficacy of oral fluid for HBV detection has been compared with serum in current research and oral fluid showed a higher sensitivity and specificity, suggesting its usefulness in epidemiological studies (67). In 2021, the efficacy of utilizing oral fluid samples for monitoring HBV mutations, genotyping, and phylogenetic analysis has been demonstrated by comparing to that of the serum samples (68).

There are many limitations for diagnosis with oral fluid samples. The low IgG concentration and the enzymes with DNAs and RNAs activity are difficulties to be solved (69, 70). To achieve higher efficiency, oral fluid collectors using mechanical friction to the mouth mucosa were designed. Although researchers presented various collection and detection methods combined with newly developed molecular techniques, improving the sensitivity and specificity of HBV markers in saliva still remains challenging.

The major problems in HBV diagnosis: Low-cost, accessibility, and simpler screening techniques are also needed to utilize in low- or middle-income zones. Among HBV‐resolved patients, some therapy can influence HBV reactivation; under these circumstances, novel biomarkers with high sensitivity are anticipated for the early diagnosis of HBV reactivation.

## 5 Immune responses of HBV-related HCC

A thorough comprehension and evaluation of the dysregulation of immune function in correlation with HBV-related antigens remains necessary, which also reminds us the significance of HBV detection. The clinical manifestations and courses of HBV-infected patients varied due to many viral and host factors. Most acute HBV infection is generally self-limited, while patients with CHB often exhibit a lifetime course. Overall, the majority of acute HBV infections are usually self-limited. HBV infection tends to result in chronic infection and life-long persistence in infants and individuals with compromised immune systems (71). There are four crucial stages of chronic HBV that determine the appropriate time for treatment: immune tolerant phase, immune active phase, immune control phase, and some might experience reactivation. However, most patients undergoing NA therapy are unable to eradicate HBV completely in the liver therefore requiring lifelong treatment. Along with HBV persistence, chronic exposure to high levels of antigen may result in over activation and tolerated function of HBV-specific immune cells (72). For example, high levels of HBsAg in circulation have a detrimental effect on HBV-specific T cells and reducing serum HBsAg could awaken these dysfunctional responses (73). Further research suggested that HBV complications including the process of oncogenesis reflect the dysregulation of related immune responses and systemically formulate them provides better insight for future clinical therapy (73).

### 5.1 T cells responses in HBV-related HCC

In 1998, Nakamoto et al. (74, 75) performed HBV transgenic mice to prove that constant impairment of HBsAg-expressing hepatocytes by HBsAg-specific cytotoxic T lymphocytes (CTLs) finally resulted in the development of HCC. Currently, the progression of HCC may be facilitated by various CD8^+^ T cells populations, such as those targeting HBV core or polymerase antigens, as well as HBV non-specific CD8^+^ T cells, effector CD8^+^ T cells, and exhausted CD8^+^ T cells.

HBV-specific CD8^+^ effector T cells can effectively suppress HBV replication by secreting pro-inflammatory cytokines and kill HBV-infected hepatocytes directly through perforin and granzyme. While, exposing to constant HBsAg and HBeAg, HBV-specific CD8^+^ T cells become exhausted and might contribute to inflammatory response, ultimately leading to HCC development (76, 77). Exhausted HBV-specific CD8^+^ T cells characterized by the expression of immune-suppressive receptors, including the programmed cell death protein-1 (PD-1), cytotoxic T-lymphocyte antigen 4 (CTLA4), T cell immunoglobulin and mucin domain 3 (TIM-3) and T cell immunoglobulin and ITIM domain (78), which was correlated with development and poor prognosis of HBV-related HCC (79). Moreover, exhausted HBV-specific CD8^+^ T cells showed impaired proliferation ability and cytotoxic functions due to upregulation of TNF-related apoptosis-inducing ligand death receptor (TRAIL-R2) (80). Dysfunctional differentiation feature of exhausted CD8^+^ T cells are not eligible to restrict HBV replication and disease progression (81). Exhausted CD8^+^ effector T cells ultimately impaired the surveillance of the adaptive immune system against tumor and resulted in tumor aggressiveness. Meanwhile, the exhaustion induced by regulatory CD4^+^ T cells (Tregs) takes a critical part in immune escape for HCC development (82). Increasing Tregs and immune-suppressive cytokines, as well as persistent high viral load are also responsible for exhaustion of HBV-specific CD8^+^ T cells (83, 84). Impaired immune surveillance of CD8^+^ T cell might also be caused by the altered anatomy structure of liver in CHB patients during fibrosis and cirrhosis. Besides, the tumor microenvironment (TME) in HCC exhibits immunosuppressive features marked by enhanced expression of pro-angiogenic factors and inhibitory ligands, resulting in impaired CD8^+^ T cell function (85).

Naive CD4^+^ T cells undergo differentiation into different effector T cell subsets. In CHB patients, the responses of HBV-specific T cells were evaluated based on different serum HBsAg levels (86). Higher expression of PD-1 on CD4+ T cells were found in individuals with HBsAg levels >50,000 IU/ml (HBs^high^) compared to those with HBsAg levels <500 IU/ml (HBs^low^). 85% and 60% of HBs^low^ patients had IFNγ^+^TNFα^+^ and IFNγ^+^ IL2^+^ CD4^+^ T cell responses respectively, compared with 33% and 13% of HBs^high^ patients. The expression of inhibitory receptors, HBV-specific CD4^+^ T cell responses, and enhancement through checkpoint inhibition were found to be correlated with serum HBsAg levels. Further insights into the immunobiology of HBV could be gained through studies that investigate the correlation between serum HBV-related antigen levels and HBV-specific immune responses. These results might help clinical prediction of immune response of CHB patients with different serum HBV-related antigen levels, which guide further treatment to prevent complications.

Th1 cells secrete pro-inflammatory cytokines to activate CD8^+^ T cells for promoting cell-mediated immunity, thus taking a key part in antitumor immunity (87). During chronic HBV infection, the Th1 dominance was replaced by Th17 cells. High Th17/Th1 ratio is related with poor overall survival in HBV-related HCC patients underwent liver resection (88). In HBV-related HCC, cytotoxic CD4^+^ T cells present similar numbers with cytotoxic CD8^+^ T cells, while they are less effective to eliminate tumor cells due to secreting less cytolytic factors such as granzyme A (GzmA) and granzyme B (GzmB). Cytotoxic CD4^+^ T cells have attributes of anti-viral and anti-tumor effects, which are carried out by activating CD8^+^ T cells and B cells. The lack of cytotoxic CD4^+^ in HCC was associated with high recurrence and poor prognosis (89).

T follicular helper (Tfh) cells are related with B cell response mainly through IL-21. In CHB patients, the response of Tfh cells to HBsAg was impaired, mainly due to CTLA-4 expression on Tregs (90). CD25^+^Foxp3^+^ Treg-like Tfh cells were found enriched in CHB patients, which showed lower IFN-γ and IL-17 production compared to the CD25^-^ Tfh cells (91). The efficiency of Treg-like Tfh cells mediating plasmablast differentiation was lower than that of the CD25^-^ Tfh cells. In patients with HCC, the levels of Tfh cells in circulation and tumor infiltration were significantly lower compared to healthy individuals and non-tumor tissues (92). The functions of Tfh cells including secreting IL-21 and facilitating B cell maturation in HCC were impaired (93). HCC-specific Tfh exhaustion may have occurred due to increased PD-1 and PD-L1 signaling (94). Inhibiting PD-1 partially restored the function of Tfh cells in individuals with stage I and II HCC.

Tregs are characterized by the expression of forkhead box protein P3 (Foxp3) (95), which is a transcriptional factor and a reliable marker of Tregs (96). Tregs maintain immune tolerance and control excessive immune activation. High levels of HBsAg in CHB patients enhanced the expansion of FOXP3^+^Treg and IL-10^+^ type-1 regulatory T cells (Tr1) through the promoted generation and functions of myeloid-derived suppressor cells (MDSC), which resulted in attenuated anti-HBV response (97). This study also discovered that even after one year treatment of tenofovir, the frequency/function of MDSC, Treg and HBsAg levels remained abnormal, despite a decrease in viral load. Hence, combination of NA and therapy targeting MDSC or inhibiting the HBsAg levels might potentially enhance the effectiveness of HBV clearance and preventing the development of HCC or other complications.

Tregs also promote the immune escape of HBV-HCC, leading to the formation of tumor thrombus in portal vein of tumor tissue (98). The increase of Tregs inhibited the specific immune response of HBV antigen and HCC tumor antigen (78). Weakening the immune surveillance, Tregs create a pro-tumor immune microenvironment. Recruitment of Foxp3^+^ T cells in HCC tumor tissue indicated development of HCC (99). Foxp3^+^CD4^+^CD25^+^CD127^low/-^ T cells showed significantly positive correlation with AFP, associated with tumor size in HBV-HCC patients (100). These results suggested that treatment inhibiting the activity of Tregs may be beneficial to patients with HCC (101).

In clinical practice, inhibiting PD-1/PD-L1 pathway enhanced the function and proliferation of HBV-specific CD8^+^T cells, which is a good therapeutic candidate (102). Simultaneous activation of OX40 and inhibition of PD-L1 in a combined therapy functionally enhanced HBV-specific CD4^+^ T cells producing IFN-γ and IL-21 (103). In 2020, therapy combining atezolizumab and bevacizumab has been established as first-line systemic treatment for patients with advanced-stage HCC (104). Tremelimumab increased the abundance of CD4^+^ and CD8^+^ T cells and elicited their activation, while reducing peripheral T cell clonality in patients with HCC (105). Blocking other co-inhibitory checkpoints to stimulate effector T cells is now paid close attention to clinically. Antiangiogenic therapy induces vascular normalization and breaks the immunosuppressive TME leading to recruitment and/or activation of effector T cells. For example, the combination of multikinase inhibitors and immune checkpoint inhibitors (ICIs) played a synergistic role and the former acted as an immune-stimulating agent by facilitating CD8^+^ T cell infiltration (106).

### 5.2 NK and NKT cells responses in HBV-related HCC

Natural killer (NK) cells take a cytotoxic part in controlling viral infection and HCC via widely activated receptors and low amounts of inhibitory receptors. However, NK cells might lose function of surveillance in the tumor microenvironment with deceased numbers, defective cytokine secretion, invalid NK cell receptors and presence of immunoregulatory cells (107). For example, the increased expression of inhibitory receptors KLRC1, TIM-3 and PD-1 and reduced expression of activating receptors such as NKG2D and 2B4 in NK cells of CHB patients suppressed secretion of IFN-γ and TNF-α (108, 109). HBsAg suppressed the expression of major histocompatibility complex class I-related molecules A and B by inducting cellular miRNAs, resulting in the inhibition of NKG2D-mediated elimination of HCC cells (110). Significant up-regulation of miR-146a in NK cells observed in patients with CHB and HCC was related to decreased cytotoxicity (111). In addition, exosomes secreted by HBV took a key part in the spread of HBV among NK cells, thus damaging the proliferation, function and survival of NK cells (108). NK cells infiltrated with lower frequency in the intra-tumoral of HCC than in the non-malignancy tissues, and CXCR6^+^ CD69^+^ liver-resident phenotype was commonest (112). More defective IFN-γ and TNF-α were produced by intra-tumoral NK cells than non-tumor NK cells. Zhang et al. discovered that CD11b^-^CD27^-^NK subgroup showed quiescent and naive phenotype with feeble cytotoxicity and less expression of IFN-γ. The high percentage of CD11b^-^CD27^-^NK cells among tumor-infiltrating NK cells explained the dysfunction of NK cells and was correlated with worse clinical outcomes (113). Natural killer T (NKT) cells with surface markers of NK are innate lymphocytes cells. They also have single invariant T cell receptors and are enriched in microvascular compartments of the liver. Two subsets of NKT cells-type I and type II showed function of inflammation and maintenance of immune tolerance (114). NKT and CD8^+^ T cells suppress HCC growth via augmentation of IFN-γ (115). During HBV infection, the production of IL-12 by CD205^+^ Kupffer cells can enhance the activation of NKT cells (116).

To enhance the cytotoxicity and specificity of NK cells against HCC, chimeric antigen receptor-NK (CAR-NK) cells have been developed. GPC3 specific CAR-NK-92 cells have been reported to have high antitumor activity against HCC xenografts expressing GPC3, suggesting a new therapy for GPC3 positive HCC patients (117). CD147-CAR-NK cells can effectively kill malignant HCC cell lines *in vitro* and HCC tumors in patient-derived xenograft mouse models safely (118). Many studies have reported that chemotherapeutic approaches can stimulate NK cells to kill HCC cells more effectively. By inhibition of AR, sorafenib elevated the IL-12A levels to enhance the cytotoxicity of NK cells in liver orthotropic xenograft mice model (119). The clinical utilization of ICIs targeting NK cells such as NKG2A inhibitors are in progress (120).

### 5.3 Kupffer cells and MDSCs responses in HBV-related HCC

Kupffer cells (KCs) may increase production of NO and TNF-α and IFN-γ to induce apoptosis of tumor, showing the effect of anti-tumor (121). HBsAg can suppress the innate immunity by blocking TLR-mediated signaling pathway and stimulating KCs to produce IL-10, which helps to sustain immune tolerance and promote anti-HBV CD8^+^ T cell exhaustion in HBV carriers (122). HBeAg from mother seems to influence the hepatic macrophages of the offspring to inhibit HBV-specific CD8^+^ T cell responses through currently unknown mechanisms (123). The chronic hepatitis and HCC progression were mediated by TNF-α, IL-6 and MCP-1 produced by hepatic macrophages, which were recruited by IFN-γ derived from CD8^+^ T cells (124). Blocking immunosuppression of PD-L1 on PKM2^+^ glycolytic macrophages might restore intrinsic anti-tumorigenic properties (125). M1 macrophages showed pro-inflammatory function via secreting IL-1α/β, IL-12, IL-18, iNOS, and TNF-α, which is prompted by LPS, IFN-γ, and GM-CSF. IL-4, IL-10, IL-13, and glucocorticoids induced M2 macrophages showed immunosuppressive function via secreting IL-10, Arg1, and PD-L1. M1 and M2 macrophages coexist in tumor microenvironment, and reprogramming macrophages polarization provides a precise strategy for anticancer therapy (126).

MDSCs are derived from myeloid progenitors and their functions and phenotypes are sculpted by diverse pathological factors. Human MDSCs are typically categorized into two subsets: granulocytic MDSCs (gMDSCs) or monocytic MDSCs (mMDSCs). Studies found that mMDSCs could inhibit T cell responses and protect liver from excessive damage during acute liver inflammation (127). However, in the context of chronic liver disease, their immunosuppressive properties enabled them to promote inflammation, affect T cells negatively and cause tissue damage through multiple mechanisms, such as inducing Tregs, expressing immune check-point molecules and depriving T cells of necessary amino acids (128). Zhong et al. found that HBsAg upregulated CCR9 on mMDSCs via activation of ERK1/2 and IL-6 and the increase of mMDSCs might negatively affect HBsAg-specific CD8^+^ T cells in young CHB patients (129). The interaction between CCR9 and CCL25 caused elevated death of HBsAg-specific CD8^+^ thymocytes by thymic homing of mMDSCs. CD11b^+^CD33^+^MDSCs infiltrated in tumor tissues of HCC patients supressed autologous CD8^+^T cell proliferation (130). MDSCs reduced the cytotoxic activity and cytokine secretion of NK cells, a process regulated by the NKp30 receptor (131). Anti-tumor therapies aiming to inhibit the immunosuppressive effects of MDSCs have been under wide development. It was reported that hepatic stellate cells (HSCs) enhanced the development and immunosuppression of mMDSCs by inducing p38 MAPK signaling (132). Treatment with p38 MAPK inhibitor disrupted the interaction between HSCs and MDSCs, leading to the prevention of HCC growth.

### 5.4 Antigen presenting cells responses in HBV-related HCC

Antigen-presenting cells (APCs), a diverse group of immune cells, process and present antigens to lymphocytes, which induce adaptive immune response. Among them, dendritic cells (DCs) take a pivotal part in initiating and shaping the adaptive immune responses by presenting antigens and producing cytokine. DCs are capable of uptaking HBV proteins, which could affect the activity of DCs negatively (133, 134). A study indicated that the generation of DCs is adversely impacted by HBeAg (135). Lan et al. found that HBeAg might decrease the secretion of IL-12 by DCs and induce the development of regulatory DCs, which assisted the persistence of HBV infection (136). The immunoregulatory function of DCs was also reported in HCC, such as promoting Tregs (137). The decreased signaling of IL-12 impaired the proliferation of T cells. Functional DCs are essential for the success of T cell-based therapy, therefore therapies targeting DCs is an attractive combination for other immunotherapy.

Liver sinusoidal endothelial cells (LSECs), unique microvascular endothelial cells, can also process circulating antigens and function as efficient APCs. They might induce tolerization of T cells mediated by PD-L1 or inducing Tregs (138). The state of LSECs is crucial for regulating the HBV-specific T cells response. HBeAg induced LSECs activation and maturation, which promoted HBV-specific CD8^+^T cells response via TNF-α signaling pathway and resulted in HBV clearance in a CHB mouse model (139). Research has shown that stimulating the NOD1 signaling pathway could also induce LSECs maturation and control HBV replication efficiently (140). In the progression of HCC, the surrounding LSECs developed structural and phenotypic modification, leading to reduced anti-tumor immune response (138). They might involve in tumor vascularization and procoagulation during oncogenesis in mice (141).

### 5.5 γδ T and mucosal-associated invariant T cells responses in HBV-related HCC

γδ T cells are the bridges of innate and adaptive immunity and assume pivotal functions in liver diseases such as HBV infection and HCC. With the levels of HBV markers elevated, the hepatic γδ T cells increased in an acute HBV infection mouse model, which is accompanied by upregulated expression of the activating marker CD69 and IFN-γ production (142). γδ T cells mainly showed antiviral function during acute HBV infection. However, these cells displayed different roles in CHB infection and studies found that the cytotoxicity of Vδ2 T cells induced by IFN-γ or TNF-α is impaired (143). γδ T cells show anti-tumor role through producing high levels of IFN-γ, TNF-α, perforins and granzymes when activation; on the other hand, IL23, IL1β and IL6 secreted from tumor cells induced the differentiation of IL-17–producing γδ T cells (γδ17 T cells) to induce vascular endothelial growth factor expression, angiogenesis, proliferation and invasion of the tumor (144). Mensurado et al. found that pro-tumoral γδ17 T cells could produce antioxidant glutathione to wear down neutrophil-derived ROS (145). In addition, PD-L1 expressing γδ T cells induce exhaustion in αβT cell by interaction with PD-1 (144). The γδ T cells possess potent cytotoxicity, tissue localization and associated with better HCC patient survival (146). γδT cells could persist long-time (>10 years) in the liver secreting superior anti-tumor cytokine and exhibit a tissue-resident memory (TRM) phenotype (CD69^+^CD49a^+^) (146). Vγ9Vδ2 T-cells, a subgroup of γδT cells, demonstrating highest TRM phenotype, achieved long local immunosurveillance for HCC (147).

Mucosal-associated invariant T (MAIT) cells are a subset of αβ T cells with both innate- and effector-like characteristics. They are enriched in mucosal tissue and liver as well as account for 1%-10% of circulating T cells (148). After TCR signals activation, MAIT cells quickly release various proinflammatory cytokines and eliminate infected cells. Studies discussing the frequency and function of MAIT cells showed conflicting results (149). Most researchers found that circulating and liver MAIT cells were decreased with impaired function or elevated exhaustion markers in the CHB patients compared to healthy donors (150, 151). In HCC patients, MAIT cells infiltrating tumor were found to be reprogrammed to a tumor-facilitating direction with corresponding transcriptomic changes and increased infiltrated MAIT cells was related with poor prognosis (152). Healy et al. engineered MAIT cells and conventional T cells with an HBV-specific TCR to kill tumor cells expressing HBV. They found that the effector ability of HBV TCR-MAIT cells might exceed those of conventional T cells, which suggested that MAIT cells could be suitable for the further research of immunotherapy targeting liver diseases (153).

### 5.6 Cytokine responses in HBV-related HCC

Cytokines are signaling molecules participated in the immune response and the balance of them significantly impacts the development of hepatitis and HCC. Compared with healthy donors, higher levels of IFN-γ, TNF-α, IL-2, IL-6 and IL-18 were found in the plasma of CHB patients (154). HBeAg could inhibit the production of IL-1β by suppressing NLRP3 inflammasome activity. However, HBcAg was found to exert the opposite effect (155). As a result, the levels of IL-1β were found to be upregulated in the serum. Released by apoptotic hepatocytes, IL-1α induced KCs-mediated compensatory proliferation therefore involved in the carcinogenesis of HCC (156). IL-6 can stimulate anti-virus immune responses against infected hepatocytes and it also participates in the development of pathological complications of CHB (157). Increased levels of IL-6 and TNF-α were found in HCC patients. Many studies discovered that IL-6 and TNF-α can induce HCC via causing inflammation and activating oncogenic transcription factor STAT3 (158, 159). Understanding the mechanism of cytokines in promoting CHB and HCC will provide us with better insight in the prevention and treatment of liver diseases.

### 5.7 The complement system in HBV-related HCC

The complement system, a pivotal part of the innate immune system, includes complement, complement receptor and regulatory protein and remain unclear in HCC progression. Using TCGA and GEO database, C1R, C6, C7 and Complement factor H-related 3 were considered prognosis-related factors in HCC outcomes (160). CFH-enriched extracellular vesicles facilitated the HCC cells proliferation by inhibiting complement-mediated cytotoxicity (161).

In patients with CHB, elevated C5a promoted hepatic stellate cell activation and inhibited its apoptosis, which showed a positive association with the extent of liver fibrosis. Aristolochic acid I activated the C3a/C3aR complement system to induce the invasion and migration of tumor cells (162). Decreased expression of C3aR/C5aR attenuates proliferation and epithelial–mesenchymal transition in HCC (163). An analysis showed that the expression of C3/C5/C3AR1/C5AR1 was correlated with the immune evasion property, suggesting the potential immune modulating part of complement proteins in HCC. Complement related protein could be utilized as potential biomarkers and therapeutic strategies for HCC. Upregulation of C1q, C3/C3a, C5/C5a in HCC is associated with tumor aggressive phenotypes, such as immune suppression, tumorigenesis, metastasis and stemness (164).

## 6 Conclusions

The review summarized the classical and modern approaches used for diagnosis of HBV. In the future, more highly sensitive novel biomarkers are expected. On the other hand, simpler, accessible, and cost-effective screening techniques are needed to apply in low- or middle-income countries. Also, we reviewed our current knowledge on various immune responses which present reprogramming properties in immune microenvironment in the progression of HBV-related HCC. The immune responses at cellular and molecular levels are highly complex and the precise mechanisms require further investigation, thus suggesting the potential strategies for the early intervention and treatment of HBV-related HCC.

## Author contributions

KL contributed to the conception of the study. WS wrote the first draft of the manuscript and KL finished the second draft of manuscript. YZ revised the final manuscript and provide funding. All authors contributed to the article and approved the submitted version.
